# Applying Convolutional Neural Network to Predict Soil Erosion: A Case Study of Coastal Areas

**DOI:** 10.3390/ijerph20032513

**Published:** 2023-01-31

**Authors:** Chao Liu, Han Li, Jiuzhe Xu, Weijun Gao, Xiang Shen, Sheng Miao

**Affiliations:** 1 School of Environmental and Municipal Engineering, Qingdao University of Technology, Qingdao 266033, China; 2 School of Information and Control Engineering, Qingdao University of Technology, Qingdao 266033, China; 3 Faculty of Environmental Engineering, The University of Kitakyushu, Kitakyushu 808-0135, Japan; 4 Department of Statistic, George Washington University, Washington, DC 20052, USA

**Keywords:** soil erosion identification, convolutional neural network, ecological restoration area, machine learning, multispectral data

## Abstract

The development of ecological restoration projects is unsatisfactory, and soil erosion is still a problem in ecologically restored areas. Traditional soil erosion studies are mostly based on satellite remote sensing data and traditional soil erosion models, which cannot accurately characterize the soil erosion conditions in ecological restoration areas (mainly plantation forests). This paper uses high-resolution unmanned aerial vehicle (UAV) images as the base data, which could improve the accuracy of the study. Considering that traditional soil erosion models cannot accurately express the complex relationships between erosion factors, this paper applies convolutional neural network (CNN) models to identify the soil erosion intensity in ecological restoration areas, which can solve the problem of nonlinear mapping of soil erosion. In this study area, compared with the traditional method, the accuracy of soil erosion identification by applying the CNN model improved by 25.57%, which is better than baseline methods. In addition, based on research results, this paper analyses the relationship between land use type, vegetation cover, and slope and soil erosion. This study makes five recommendations for the prevention and control of soil erosion in the ecological restoration area, which provides a scientific basis and decision reference for subsequent ecological restoration decisions.

## 1. Introduction

Soil is the center of terrestrial ecosystems. In recent years, the rate of soil erosion has been accelerating due to global climate change, land cover change, and land use change [1,2]. It poses a threat to the ecological environment and human health [3], and causes serious social and economic problems [4]. Soil erosion is a complex process [5]. In the past decades, severe soil erosion models have been developed to characterize soil erosion. However, most of these models are linear, usually derived from specific scientific questions, and are only applicable to specific areas [6].

Currently, many ecological restoration projects (ERPs) have been implemented, including soil improvement, afforestation, and grass planting, to reduce soil erosion. Among them, plantation forest is a typical ecological restoration plan [7,8]. Different from natural forests, plantation forests have simple structures, poor understory vegetation, and poor ecological stability [9,10,11], so their soil retention capacity is relatively weak. However, traditional erosion models are built based on natural forests without taking into account the differences between planted and natural forests. Therefore, it is not reasonable to use traditional soil erosion models in this study because they may lead to biased or inaccurate prediction results in the study area. In recent years, many machine learning models have been widely used in soil research [12], such as soil frost depth prediction [13], soil salinization analysis [14], soil erosion [15], and soil microbial assessment [16]. However, there are few types of research on soil erosion based on machine learning, especially in coastal ecological restoration areas. Machine learning techniques could express linear or nonlinear relationships between soil erosion and its erosion factors [17], and thus estimate soil erosion.

The southern catchment area of Xuejiadao is located in a coastal area with important ecosystems and natural resources. However, influenced by sea level rise (SLR) and frequent hurricanes, the soil in the southern part of Xuejiadao has been eroded to varying degrees. In order to restore the regional ecosystem, a large number of planted forests have been planted in the area. The aim of this study is to apply the convolutional neural network (CNN) system model to identify the soil erosion status of ecological restoration areas (especially plantation forests) through high-resolution image data collected by unmanned aerial vehicles (UAVs) and field sampling data, so as to assess and analyze the soil erosion intensity in the southern catchment area of Xuejiadao in the coastal area. The main contributions of this paper are as follows.

1. This paper applies the CNN system model to identify the soil erosion intensity in ecological restoration areas (mainly plantation forests), providing an advanced research method for the study of soil erosion in ecological restoration areas (mainly plantation forests).

2. This study applies the multispectral data collected by the UAV in a smaller scale area to study soil erosion, proving that the UAV can be applied in the field of soil erosion research, and can improve the resolution of the collected data, making the identification results more accurate.

3. This paper provided a targeted research method for the study of soil erosion in the forest understory of ecological restoration areas, and provided a template for related research on data collection, factor analysis, and prevention and control management.

The remainder of this study is organized as follows. Section 2 presents the work related to soil erosion research and the relevant techniques used in this study. Section 3 focuses on the introduction of the findings of the study area, the data collection process, the index calculation process and the research methodology. Section 4 provides the experimental results with a spatial analysis, in addition to a discussion and recommendations on soil erosion in the study area, and Section 5 is the conclusion of this study.

## 2. Related Works

Soil erosion is a highly complex natural process [6] and one of the most important ecological problems in the world [18]. It causes soil degradation, water pollution, and so on, which seriously affects local economic growth. Many scholars have performed much research to analyze the soil erosion problem. Typical soil erosion studies are carried out through field observations, experimental manipulations and soil erosion simulations. Traditional experimental methods are easy to implement in small-scale studies [19], but are expensive and difficult to apply in large-area studies. Therefore, model simulation has become a convenient method for soil erosion studies. Wischmeier and Smith proposed the famous universal soil loss equation (USLE) [20], which was successfully applied in the eastern part of the United States to predict the amount of soil loss. However, the USLE model is applicable to slopes with slopes ranging from 0° to 7°. To improve the unreasonable analysis method of USLE and increase the accuracy and scope of the model, based on USLE, Renard [21] developed the revised universal soil loss equation (RUSLE) [22] model. However, it still follows the basic framework of USLE, and it is still an empirical statistical model and only applicable to plain and gently sloping areas. In addition, there is the Water Erosion Prediction Project (WEPP) [23], and the Revised Wind Erosion Equation (RWEQ) [24]. However, these models are mostly derived from specific scientific problems and may be valid for specific situations [6]. The differences between planted and natural forests are not considered in the soil erosion process, so it is not reasonable to use these models in this study.

The extensive application of remote sensing (RS), global positioning system (GPS), and geographic information system (GIS) technology has promoted the development of soil erosion research. By using “3S” technology, researchers could efficiently and accurately establish a regional soil erosion information system and realize dynamic monitoring of soil erosion [25]. In addition, the development of UAV technology in recent years has provided a simple tool for acquiring high spatial resolution data at the centimeter level. High-precision and high-resolution Digital Elevation Model (DEM) images acquired by UAVs are widely used in soil erosion research [26,27] and disaster risk assessment research [28]. Moreover, a large number of studies leveraged UAV multispectral data to identify land use types [29] and vegetation types [30,31]. In addition, UAV images are also used to monitor soil erosion [32] and vegetation growth [33]. UAVs have been widely used in various industries because of their convenience and ability to obtain high-precision remote sensing images [34].

With the development and maturity of big data technology and high-performance computing, artificial intelligence techniques represented by deep learning modeling have made great achievements in target detection, image recognition, machine translation, and many other fields. Furthermore, deep learning has received increasing attention from experts in remote sensing and environmental sciences. Compared with traditional machine learning (such as support vector machine, random forest, etc.), the advantage of deep learning is that it does not require manual extraction of required features from unstructured data like images and texts and could save a lot of manpower. CNN is one of the most representative algorithms in deep learning to consume images and videos. In recent years, CNNs have been widely applied in remote sensing image classification [35], road extraction [36], building extraction [37], water extraction [38], and other fields. For instance, Chen [39] classifies PolSAR images based on the improved CNN model and achieves great performance with a small number of training samples. Kavita [40] also applied CNN to classify LULC in the Indian pine dataset, and the classification accuracy reached 97.58%. The results show that CNN is effective in the practical application of unstructured data and even with a small dataset.

In summary, most research within the field of soil erosion research has been conducted based on traditional soil erosion models. Traditional soil erosion models express a linear mapping between erosion factors and soil erosion. Actual soil erosion is the result of the interaction between the erosion factors and is a complex nonlinear mapping relationship. In addition, traditional soil erosion models are mostly based on satellite remote sensing, which is susceptible to atmospheric influence and the current technology has not yet fully solved the atmospheric correction problem; the resolution of satellite remote sensing images is low. The use of traditional soil erosion models can no longer accurately represent the soil erosion conditions in the understory of ecological restoration areas (especially in plantation forest areas). In the study area, this study uses multispectral images collected by UAV as research data, which can provide higher resolution data; applying the CNN model to identify the soil erosion intensity in the ecological restoration area (mainly plantation forest) can make the identification more accurate.

## 3. Materials and Methods

### 3.1. Study Area

**Figure 1:**
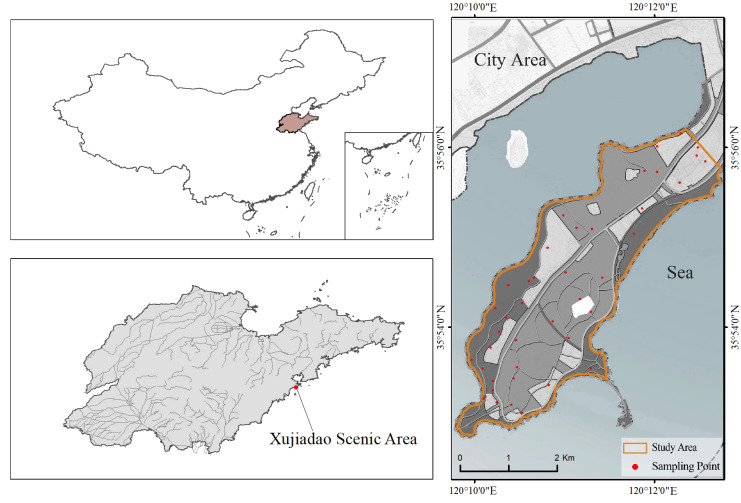
The sketch map of Xuejiadao.

The southern part of Xuejiadao is located in the northeast of the west coast new area of Qingdao, China. Its geographic coordinate information and boundary are shown in Figure 1. The whole research area is a long and narrow peninsula surrounded by the sea on three sides extending from northeast to southwest. It is related to land in the northeast, Tangdaowan in the south, and the Yellow Sea in the east. The length of the study area from northeast to southwest is about 6.2 km, the average width is 1.6 km, and the total area is 751.02 hm${}^{2}$. The study area belongs to a low hilly area, and the overall terrain is high in the middle and low around. The elevation is between 0 and 100 m, with a high slope in the middle. The soil is mostly brown loam, which thickens gradually from high to low with the fluctuation of the terrain. Affected by seawater intrusion, soil salinization is serious in the study area. The study area belongs to the warm temperate semihumid monsoon climate, which is directly affected by the marine monsoon, ocean currents and water masses, with moist air and abundant rainfall [41]. The average annual temperature of the study area is 12.6 °C, and the average annual precipitation is 732.1 mm, which is concentrated from June to September, and there are often torrential rains. There are no large rivers in the study area and some artificial wetlands in the northwest. The land cover types are mainly construction land, grassland, bare land, planted forest and so on. The planted forests are mostly *Pinus thunbergii* and *Quercus acutissima*, with a single structure and low understory vegetation cover.

### 3.2. Data Collection

Data collection is the fundamental step of scientific analysis. The data collection quality directly affects the value of the data analysis results. The data used in this study are primarily divided into basic data and sample data. The basic data include geographic location, multispectral, topographic, rainfall, soil, and other data. Basic data are mainly obtained through drones and third-party platforms. Sample data were obtained by field sampling.

#### 3.2.1. Basic Data

UAV data were collected by the research team in the study field from September 2021 to October 2021. The UAV model is DJI Phantom 4 Multispectral (P4M), with an aerial accuracy of about 0.05 m. The UAV is equipped with an F2.2 aperture lens, six 2.9-inch 2-megapixel CMOS sensors and an electronic global shutter. In the process of data collection, the flight altitude of the UAV was set at approximately 100 m above the ground, the heading overlap rate was set at 80%, and the side overlap rate was set at 70%, to guarantee the integrity of subsequent images. The flight time is selected from 10:00 am to 2:00 pm local time to reduce the spectral image of the direct sun angle. The duration of a single flight is about 20 min, and the total flight time in the research area is about 20 h. The shooting route is “bow” shaped, and the total flight distance is about 460 km, taking approximately 148,680 photos as detailed in Table 1. When the spatial resolution of the UAV image is 0.05 m, the size of the panoramic image of the study area is large, which leads to low efficiency of computer modeling. However, when the spatial resolution of the image is 0.1 m, not only can the rich spectral information of the UAV image be well preserved, but the modeling efficiency of the computer is also high. Therefore, this study uses ArcGIS 10.8 to resample the resolution of the single-band image to 0.1 m.

**Figure 2:**
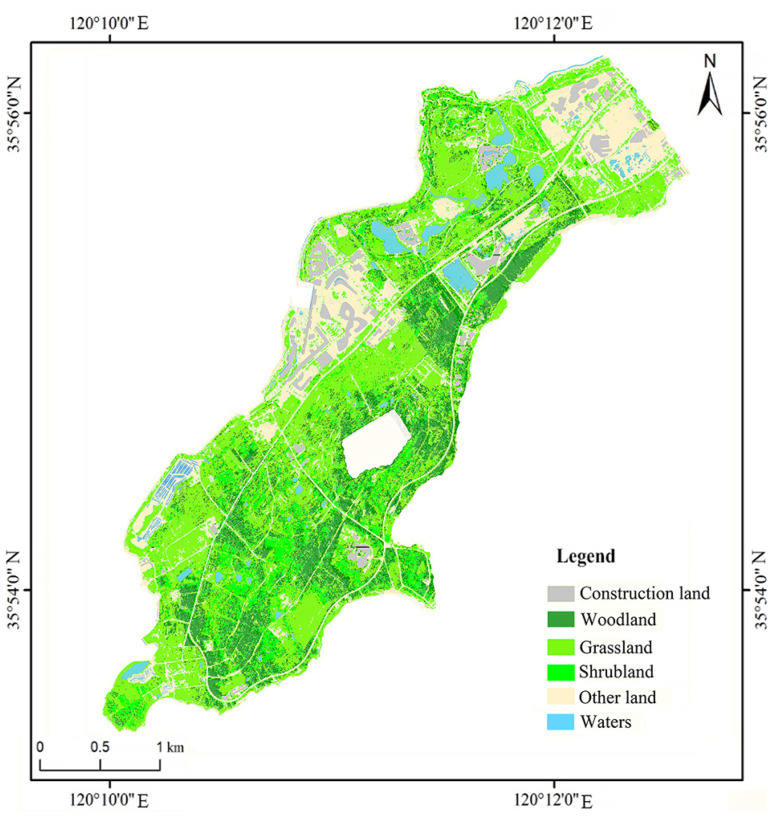
Land use classification map of southern Xuejiadao.

The data of elevation obtained by the multispectral UAV are actually the digital surface model (DSM) data. In this study, DSM is processed and the main buildings and trees on the surface are filtered out through intelligent human–computer interaction editing to obtain the DEM of the study area. The data of soil mainly come from the China soil database (http://vdb3.soil.csdb.cn/ (accessed on 27 January 2023)), including sand content, silt fraction, clay fraction, organic carbon content, etc. The data of rainfall are obtained from the Huangdao district meteorological station in Qingdao, China. The data of land use (Figure 2) are interpreted based on UAV images, regional topography, etc.

#### 3.2.2. Sample Data

**Figure 3:**
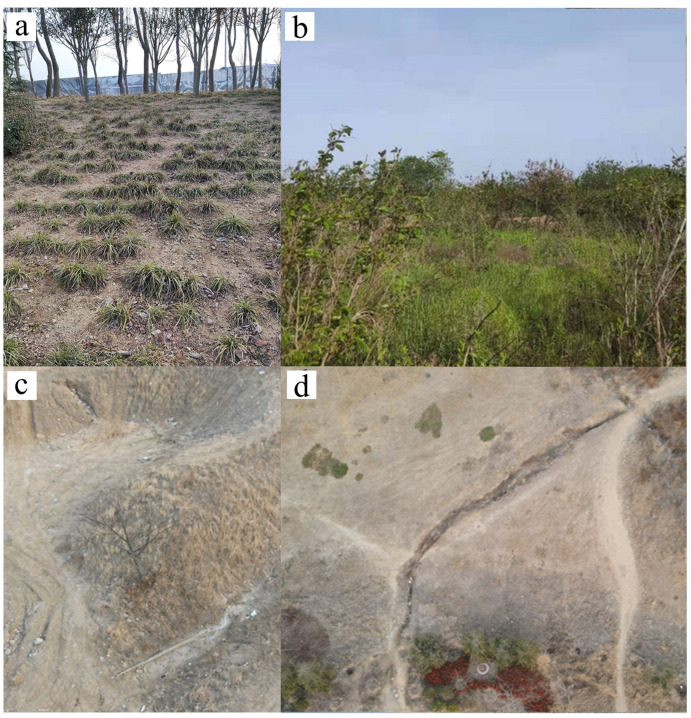
Soil erosion status in the south of Xuejiadao in 2022: (**a**) artificial understory grassland; (**b**) wild grassland; (**c**) abandoned landfills; and (**d**) wasteland erosion gully.

To explore the actual situation of soil erosion in the study area, field investigation and data collection are carried out by using UAV and handheld GPS, etc. Sampling points are selected uniformly and randomly in the study area (Figure 1 and Figure 3). Due to the different conditions of the ecological environment, meteorology, economy and soil properties in different regions, the critical threshold of soil erosion is not the same. Combined with relevant studies, this study divides the soil erosion intensity in the study area into three categories according to the measured situation of the region: slight, light, and moderate. With the support of ArcGIS, the soil erosion intensity types of the sampling sites are marked.

### 3.3. Index Calculation

#### 3.3.1. Rainfall Erosivity Factor

The empirical formula of rainfall as a variable proposed by Wu [42] in 1994 was used to calculate the parameters of the rainfall erosivity factor (R) in this study. R is expressed as an annual average rainfall erosivity index, which indicates the kinetic energy of rainfall (MJ·hm${}^{-2}$) at the maximum 30 min rainfall intensity (mm·h${}^{-1}$) in a year. The annual and monthly statistics of rainfall obtained from weather stations around the study area are used to calculate the rainfall in the study area, and the R factor in 2021 is obtained by using Equation (1),

(1) $$R=\sum_{n=1}^{12}0.0125P_{i}^{1.6295},$$

where R represents the rainfall erosivity factor, MJ·mm·hm${}^{-2}$·h${}^{-1}$·a${}^{-1}$. $P_{i}$ is the monthly rainfall, mm. Due to the small geographical scale of the study area, there is no significant difference in the spatial distribution of rainfall erosivity. According to the regional annual rainfall in 2021, R is 183.12 MJ·mm·hm${}^{-2}$·h${}^{-1}$·a${}^{-1}$.

#### 3.3.2. Soil Erodibility Factor

Soil erodibility (K) refers to the ability of soil to be easily damaged by erosion, and it is an important indicator to use when evaluating the sensitivity of soil to erosion [43]. K is determined by its physical and chemical properties. In this study, soil data information is obtained from the above soil database, and the soil erodibility factor is obtained by using the K calculation Formula (2) [44]

(2) $$\begin{array}{c} K=\frac{1}{7.59}\left\{0.2+0.3e^{\left[-0.0256\mathrm{SAN}\left(1.0-\frac{\mathrm{SIL}}{100}\right)\right]}\right\}\times {\left(\frac{\mathrm{SIL}}{\mathrm{CLA}+\mathrm{SIL}}\right)}^{0.3}\times \\ \left[1.0-\frac{0.25C}{C+e^{\left(3.72-295C\right)}}\right]\times \left[1.0-\frac{0.7\mathrm{SNI}}{\mathrm{SNI}+e^{\left(-5.51+22.9\mathrm{SNI}\right)}}\right], \end{array}$$

where SAN is the sand fraction, %; SIL is the silt fraction, %; CLA is the clay fraction, %; C is the organic carbon content, %; SNI = 1 − SAN/100.

**Figure 4:**
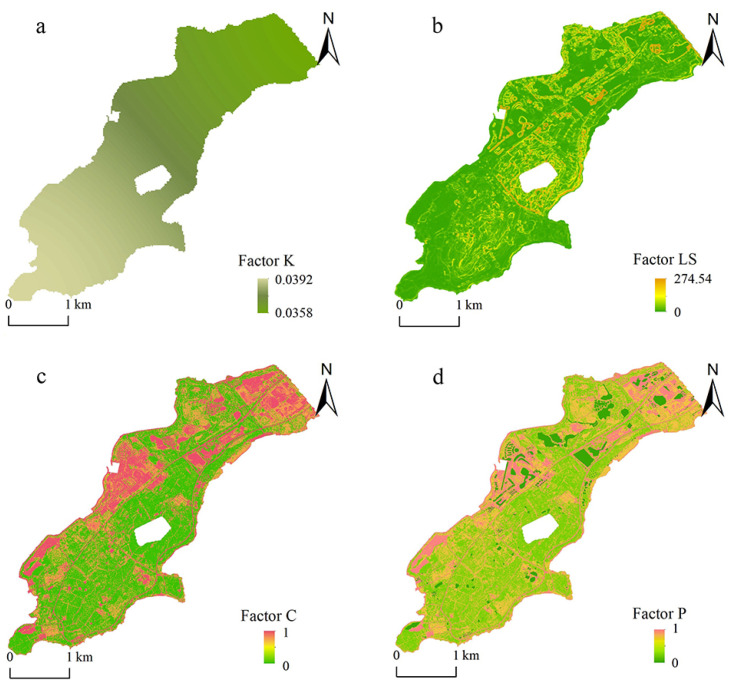
The spatial distribution of K factor (**a**), LS factor (**b**), C factor (**c**) and P factor (**d**) in the study area.

The geographical space of the study area is small. Soil types are mainly coarse bone soil and leached soil. In this study, the inverse distance weight tool of ArcGIS 10.8 is used for interpolation analysis, to obtain the distribution of the K value in the study area (Figure 4). The spatial distribution of soil erodibility factors showed little difference as indicated by the figures.

#### 3.3.3. Topographic Factor

Topographic factors (LS) are one of the important influencing factors of soil erosion. Topography directly determines the manifestations of soil erosion and the ability of runoff to wash away. Topographic factors include slope factors and slope length factors. According to domestic research results, this study chooses different formulas to calculate the slope steepness factor. We have

(3) $$S=\left\{\begin{array}{cc} 10.8\sin\theta +0.03 & \theta <5^{\circ }\\ 16.8\sin\theta -0.50 & 5^{\circ }\leq \theta <10^{\circ }\\ 21.9\sin\theta -0.96 & \theta \geq 10^{\circ }, \end{array}\right.$$

where θ is the slope (${}^{\circ }$), *S* is the slope steepness factor.

In this study, the confluence area method [45] is adopted to calculate the slope length factor. The formula is as follows,

(4) $$L_{i}=\left\{\begin{array}{cc} \frac{{\left(A_{\mathrm{out}}\right)}^{\left(m+1\right)}-A_{\mathrm{in}}^{\left(m+1\right)}}{{\left(\Delta x\right)}^{2}\cdot {\mathrm{NCSL}}_{m}^{m}{\left(22.13\right)}^{m}} & \lambda_{\mathrm{out}}\neq 0\\ 0 & \lambda_{\mathrm{out}}=0 \end{array}\right.,$$

where $L_{i}$ is the slope length factor of the *i*th grid; $A_{\mathrm{in}}$ and $A_{\mathrm{out}}$ are the catchment areas at the entrance and exit of the grid respectively (m${}^{2}$); Δx represents raster resolution in meter; *m* is slope length index; and NCSL represents the noncumulative slope length related to the flow direction at the entrance and outlet of the grid in meters.

The slope length index changes with the gradient. Its value refers to the research results of Liu [46]. The values of *m* are as follows,

(5) $$m=\left\{\begin{array}{cc} 0.2 & \theta <0.5^{\circ }\\ 0.3 & 0.5^{\circ }\leq \theta <1.5^{\circ }\\ 0.4 & 1.5^{\circ }\leq \theta <3^{0}\\ 0.5 & 3^{\circ }\leq \theta \end{array}\right.,$$

where θ is the slope.

According to the terrain of the region, we set the threshold of the convergence area as 10,000 m${}^{2}$. The slope steepness factor and the slope length factor were calculated by LS software.

#### 3.3.4. Cover Management Factor

Vegetation is an important factor affecting soil erosion. According to relevant studies, the soil erosion rate decreases with increasing vegetation coverage [47]. The cover management factor (C) reflects the protection of the soil by vegetation and its inhibitory effect on soil erosion. Based on the method of Cai [44], combined with the actual situation of vegetation cover in the study area, this study adopts the revised calculation formula to establish the mathematical relationship between vegetation cover and C, and estimates the value of C. We have

(6) $$C=\left\{\begin{array}{cc} 1 & \mathrm{FVC}\leq 0.1\\ 0.6508-0.34361g\mathrm{FVC} & 0.1<\mathrm{FVC}\leq 0.783\\ 0 & \mathrm{FVC}>0.783 \end{array}\right..$$

FVC remote sensing inversion is mainly realized by normalized difference vegetation index (NDVI) [48]. Its calculation formula is as follows,

(7) $$\mathrm{FVC}=\frac{\mathrm{NDVI}-{\mathrm{NDVI}}_{\mathrm{soil}}}{{\mathrm{NDVI}}_{\mathrm{veg}}-{\mathrm{NDVI}}_{\mathrm{soil}}}$$

(8) $$\mathrm{NDVI}=\frac{\mathrm{NIR}-\mathrm{Red}}{\mathrm{NIR}+\mathrm{Red}},$$

where ${\mathrm{NDVI}}_{\mathrm{veg}}$ is the value of NDVI statistical results when the cumulative probability is 95%; ${\mathrm{NDVI}}_{\mathrm{soil}}$ represents the minimum value of bare soil pixel; NIR represents the reflectance of the near-infrared band of multispectral image; red represents the reflectance of the red band of the multispectral image.

In this study, FVC was calculated by ENVI 5.3. According to Formula (8), the map algebra operation tool of ArcGIS 10.8 is used to obtain the spatial distribution map of C. The result is shown in Figure 4.

#### 3.3.5. Conservation Practice Factor

The conservation practice factor (P) is the most intuitive factor used to test the effectiveness of soil and water-conservation measures and evaluate the degree of soil and water loss [49]. The P factor is related to the land use type of the underlying surface. According to domestic and foreign studies [50], in the study of P factor calculation method, the assignment method is mainly used. The assignment of the P value (Table 2) in this study is based on relevant studies and the survey. In this study, according to the land use types of the research area obtained by remote sensing interpretation, the P factor of the research area was obtained by using the raster calculator of ArcGIS, as shown in Figure 4.

### 3.4. Convolutional Neural Network Model

**Figure 5:**
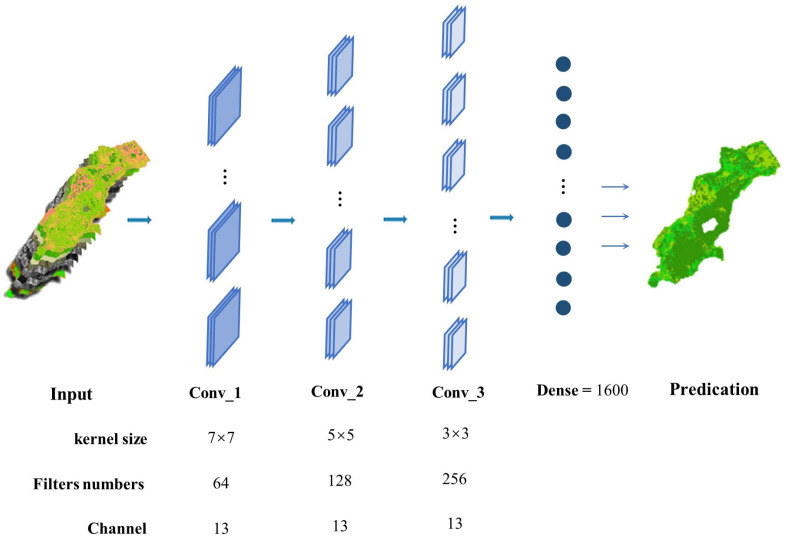
CNN frame diagram.

CNN refers to a class of neural network models with convolutional structures. CNN is mainly composed of several different types of layers: input layer, convolutional layer, pooling layer, fully connected layer and output layer, as shown in Figure 5. Among them, the convolution layer is the core layer of CNN construction because the information extraction from the input image is mainly accomplished by the convolutional layer with a series of small reception fields. The pooling layer is mainly used to reduce the size of feature maps after convolution. It can reduce the model complexity by compressing data dimensions and reducing the number of parameters. The pooling layer can also effectively prevent the overfitting phenomenon of the model. With the effect of the convolution layer and pooling layer, CNN could extract input image features more efficiently than traditional feedforward neural networks with inductive bias introduced by convolutional kernels. This study uses CNN to classify soil erosion intensity in the southern part of Xuejiadao.

In this study, remote sensing images of UAV are resampled to obtain images with a resolution of 22,990 × 20,009. The image is then divided into an average of 432,063 pixel blocks as raw data. The pixel block size is 20 × 20. The images are marked according to the results of the fieldwork for this study. Multispectral high-resolution image features will lead to a larger CNN model, more parameters, and more difficult training. Therefore, in this study, the CNN model uses a large scale convolution operation instead of pooling layer operation. The model consists of one input layer, three convolutional layers, one fully connected layer, and one output layer. The input image size is 20 × 20, and the number of channels is 13. It is used to receive five soil erosion factors, three RGB channels and five UAV multispectral images.

Because the convolution operation features are extracted layer by layer, the more detailed features, that is, smaller and more convolution nuclei are required. Therefore, the size of the convolution kernel is usually set to decrease layer by layer, and its number is set to increase layer by layer. The parameters of the three convolutional layers in this experiment are 64 convolutional kernel (with a size of 7 × 7), 128 convolutional kernel (with a size of 5 × 5) and 256 convolutional kernel (with a size of 3 × 3). This study does not apply common pooling functions in the CNN model; instead, we set the convolution stride as 2 which can decrease parameters for each hidden layers. The advantages of using large stride convolution are decreasing calculation cost and avoiding the overfitting problem. The ReLU function is applied as the activation function. The output images of the convolutional layer are then flattened and input into the fully connected layer, including 1600 nodes. The final output layer contains three neuronal nodes and uses the softmax activation function to output the predicted probabilities of three different soil erosion intensities.

In the original data set, 64% (7680) of the pixel blocks are randomly selected as the training dataset, 16% (1920) of the pixel blocks are selected as the verification set, and 20% (400) of the pixel blocks are selected as the test set. The validation set is used to further optimize the model and determine the selection of hyperparameters, and the performance of the model is reported according to the evaluation results of the test set. To minimize the error between the predicted and true values, cross-entropy is chosen as the loss function.

The Adam optimizer is one of the most popular optimizers in the field of machine learning in recent years. The Adam optimizer is easy to fine-tune and has strong adaptability. Therefore, in this study, the Adam optimization algorithm which adaptively adjusts learning rate is used to train the model. The Adam learning rate is set as 0.001, the exponential decay rate of first-order moment estimation is set as 0.9, the exponential decay rate of second-order moment estimation is set as 0.999, and the fuzzy factor is set as $10^{-8}$.

In addition, this experiment is based on Python and the TensorFlow deep learning framework. The model training and evaluation are conducted in the machine learning workstation, configured with Core i7-11700k CPU, 64GB RAM, and Windows 10 operating system.

## 4. Results and Discussion

This section applies the CNN model to study the regional soil erosion situation. The results of the CNN model are compared with the results of the Revised Universal Soil Loss Equation (RUSLE) model proposed by Renard [51], which is widely used in the field of soil erosion and is based on a linear fitting approach. In addition, in order to verify the superiority of CNN models, the SVM and feedforward neural network (FNN) modelings are selected for comparison in this study. According to the final recognition results, the spatial distribution of soil erosion in the study area is described. In addition, this section analyzes the relationship between soil erosion and single factors such as land use type, vegetation cover and slope, and proposes appropriate suggestions for soil erosion prevention and control based on the analysis results.

### 4.1. Modeling Result

**Figure 6:**
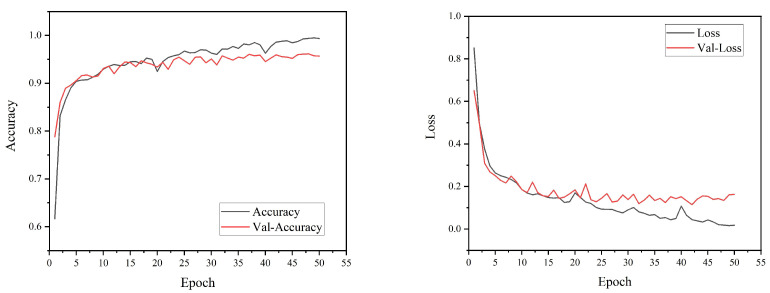
Accuracy and Loss Curves of CNN Model Training.

As seen from the accuracy images in Figure 6, with the increasing number of training rounds, the accuracy of the training set rises rapidly and then increases steadily, and the accuracy of the validation set stabilizes after a rapid increase and does not decrease, which indicates that the model is overfitting. In addition, it can be seen from the loss images that the training set converges quickly and then decreases steadily, the validation set converges quickly and tends to a steady state of convergence, and there is no significant increase in the loss value after decreasing during the training process, which indicates that there are no local minima in the model during the training process. After 50 iterations, the performance of the validation set was relatively stable, with essentially no change in accuracy or loss. At this point, the accuracy of the CNN test set was relatively high.

**Figure 7:**
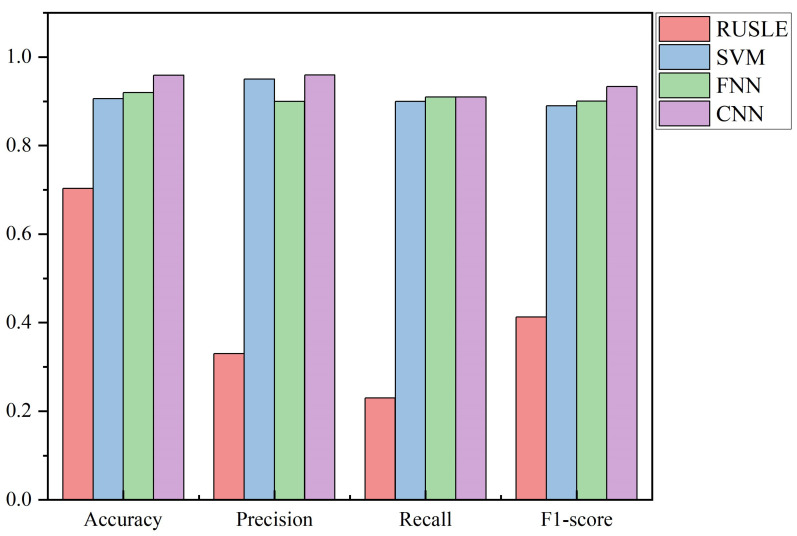
The classification evaluation index results of the three models.

RUSLE is currently one of the most commonly used models in the field of soil erosion research and is used as a baseline for this study. SVM is a supervised machine learning algorithm for statistical classification and regression analysis [52]. SVM is currently one of the most widely used machine learning algorithms [53], and can deal with the complex and nonlinear relationships between independent variables and dependent variables [54]. FNN is the first type of simple artificial neural network invented in the field of artificial intelligence. Its structure is simple and widely used [55]. In this study, SVM, FNN, and CNN algorithms are compared to verify the accuracy and effectiveness of CNN. As can be seen from Table 3, the accuracies of the RUSLE, SVM, FNN, and CNN test sets are 70.33%, 90.63%, 92.00%, and 95.90%, respectively. Due to the random selection of sample data, the number of various samples is not balanced, and precision alone does not measure the results well. To better evaluate the classification recognition effect of each model, the recall, accuracy, and F1-score of each model (RUSLE, SVM, FNN, and CNN) for each classification are calculated, as shown in Table 3. As can be seen from Table 3, the RUSLE model has zero recall for the identification of mild and moderate erosion, and cannot accurately classify the soil erosion intensity in the study area. This is because the RUSLE model is a linear model that cannot represent the complex soil erosion relationships in ecological restoration areas. From Figure 7, the CNN model achieves the most favorable results in this study.

**Figure 8:**
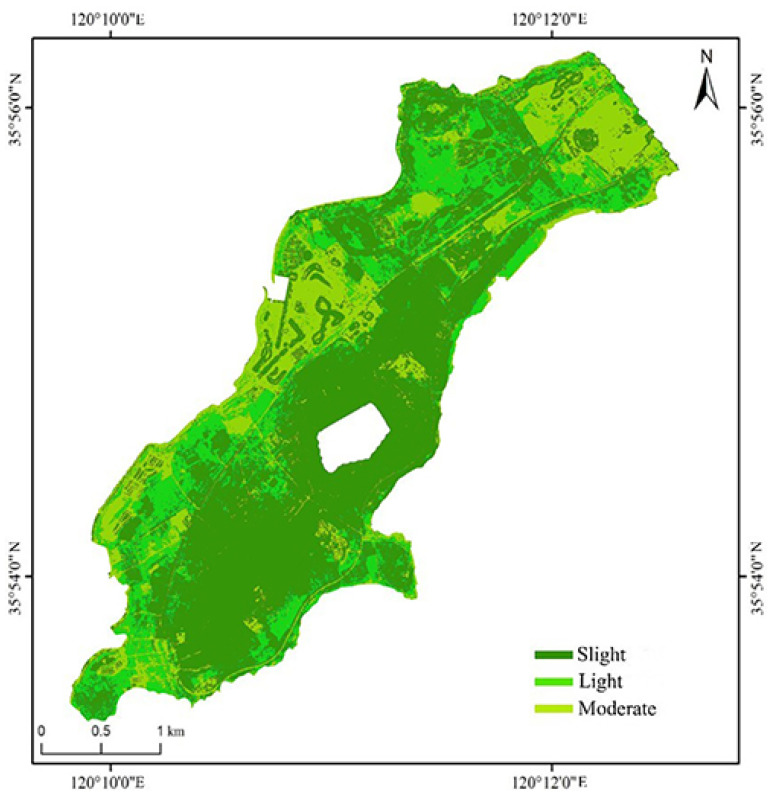
Spatial distribution map of soil erosion intensity.

By comparing the output results of the RUSLE, SVM, FNN and CNN models, this study selects CNN as the model for predicting soil erosion in ecorestoration and studies the spatial distribution rules of soil erosion in the studied area by the CNN model, as shown in Figure 8.

### 4.2. Spatial Analysis

#### 4.2.1. Correlation Analysis between Land Use and Soil Erosion Intensity

The intensity of soil erosion varies with different land use types (Table 4). The grassland area in this area is relatively large, mainly showing slight erosion and light erosion, accounting for 85.31%. Although the area of bare land is slightly smaller than that of grassland, accounting for 21.73%, the intensity of soil erosion is significantly higher than that of bare land use types, with moderate erosion accounting for 58.9% of its area. The moderately eroded area of bare land reaches more than half of the total moderately eroded area. The area of woodland and shrubland in this area accounts for 28.64% of the total area, and most of them are dominated by slight erosion. The slight erosion of woodland and shrubland accounted for 87.56% and 86.64%, respectively. This is mainly because the roots and stems of trees and shrubs have a larger range of folding, and the roots are deeper, which could consolidate the soil and enhance erosion resistance and resistance of the soil. However, there are also places of moderate erosion in the woodland, which is due to the single structure of planted forest and the existence of bare ground. In addition, the construction land and water area account for 7.35% of the study area, and there is almost no soil erosion problem. Related soil and water conservation measures have been adopted in the construction area, and there is almost no bare surface.

#### 4.2.2. Correlation Analysis between Vegetation Cover and Soil Erosion Intensity

**Figure 9:**
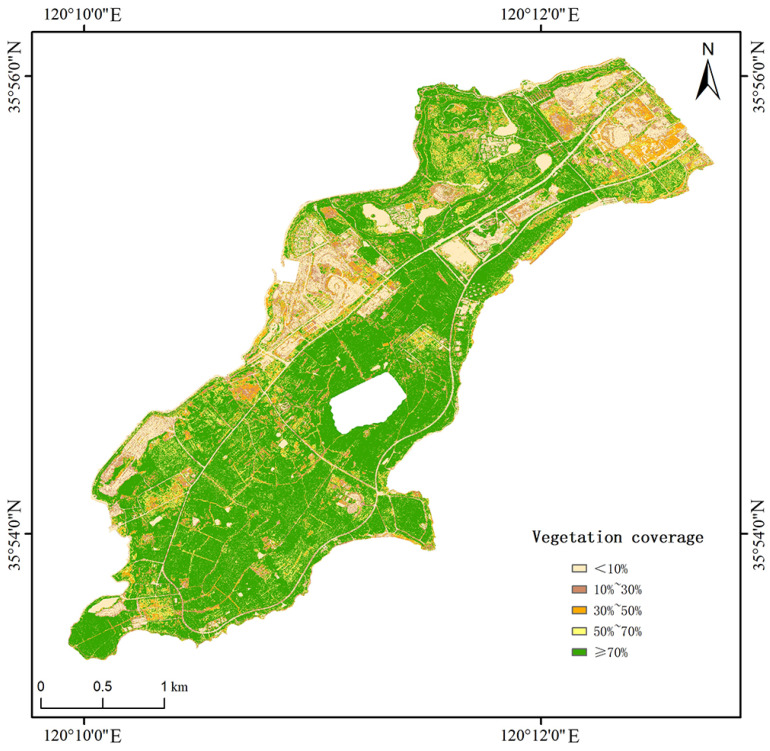
Vegetation coverage in the study area.

Vegetation cover is an essential index used to study soil erosion. It is very significant to analyze the change of soil erosion intensity under different vegetation covers in the study region. According to past studies, the vegetation coverage in the southern part of Xuejiadao could be divided into five levels: high coverage (≥70%), medium high coverage (50∼70%), medium coverage (30∼50%), medium low coverage (10∼30%), and low coverage (<10%) (Figure 9).

**Figure 10:**
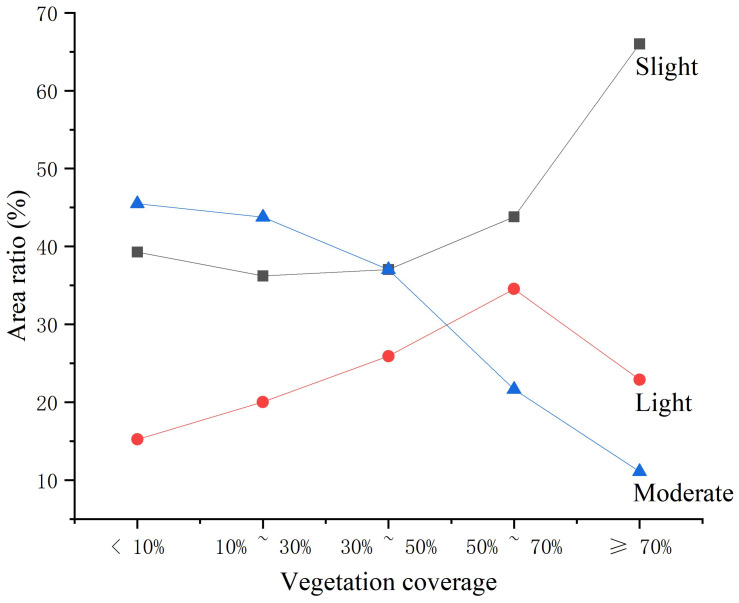
Relationship between vegetation cover and soil erosion intensity.

In this study, the spatial data of vegetation coverage and soil erosion intensity are superimposed. As can be seen from Figure 10 and Table 5, there is a high correlation between vegetation coverage and soil erosion intensity in the study area. The area of low coverage in the study area accounts for 16.64%, which is mainly focused in construction land, waters, and bare land. The vegetation coverage of construction land and waters is almost zero, and soil erosion hardly occurs. The vegetation coverage of bare land is low, and the rainfall directly washes the soil so that soil erosion is more serious. The intensity of soil erosion decreases as the increase of vegetation coverage in medium low coverage, medium coverage and medium high coverage. The proportion of high coverage is as high as 57.2%, and the proportion of slight erosion is 66%. This is because there is a large planted forest in the study area, and the closure degree of vegetation is high. Plant leaves and deciduous branches could effectively reduce the kinetic energy of raindrops, intercept surface runoff, increase soil infiltration, and thus reduce soil erosion. However, due to the incomplete structure of some understory vegetation, the soil and water conservation function of the vegetation community is reduced, and there are still areas of moderate erosion in the high vegetation coverage area.

#### 4.2.3. Correlation Analysis between Slope and Soil Erosion Intensity

The terrain of the study area is relatively flat. The slope is reclassified in ArcGIS according to the Technical Regulations of Land Use Status Investigation. The slope is graded into five levels: ≤2°, 2∼6°, 6∼15°, 15∼25°, and >25°. The slope grade map overlaps with the spatial distribution map of soil erosion intensity. This study superimposes the slope raster map and the spatial distribution map of soil erosion intensity to obtain the distribution information of soil erosion intensity of different slopes in the study area, and then analyzes the relationship between slope and soil erosion intensity.

It could be roughly seen from Table 6 that most slopes in the study area range from 0° to 15°, the terrain is relatively slow, and the soil erosion is mainly slight and light. Except ≤2°, the intensity of soil erosion in other regions increases with the increase of slope. This is because most of the areas ≤2° are village ruins, the surface vegetation is poorly developed, and the rainfall directly washes the soil, resulting in surface runoff. Even if the slope is gentle, it will cause soil loss. The proportion of moderate erosion in the area >25° is the largest, reaching 51%. This area is dominated by construction land, which is seriously affected by human activities. It has more microtopography, and temporary mounds of loose soil, so it is more vulnerable to erosion. This region is a key area for future prevention and control so that related soil and water conservation strategies are required to propose and implement. In addition, moderate erosion generally increases with the increase of slope. In general, slope is positively correlated with soil erosion intensity.

### 4.3. Discussion

The study area is seriously affected by salinization, and the plant resources are relatively single and poor. Although ecological forests have been planted locally to improve the ecological environment, a complete ecological protection system was not formed overall. The understory vegetation in the study area is small and a large amount of the ground is exposed. At the same time, coastal areas are often affected by heavy rains, which aggravate the soil erosion problem in the early stage of coastal ecological restoration. In addition, unreasonable construction activities also aggravate the occurrence of soil erosion. At present, research on soil erosion has been very extensive. However, research on soil erosion in areas with little understory vegetation and salinization problems is still limited.

This study applies the CNN system model to identify the erosion intensity of ecological restoration areas (mainly plantation forests) based on high-resolution images collected by UAVs, and compares its identification results with the baseline model (RUSLE model); in addition, to verify the superiority of the CNN model, the CNN results are compared with SVM and FNN. The results show that CNN could better express soil erosion [56]. Based on the results of the CNN model, this study analyzes the spatial distribution characteristics of soil erosion intensity in the south of Xuejiadao. The results show that soil erosion in the region of study is mainly slight erosion and light erosion. Slight erosions are located to the south and east of the study area. The reason for this trend is that the planted ecological forest reduces rainfall erosivity and blocks surface runoff, thus reducing soil loss [57], indicating that the method of increasing vegetation coverage in the study area has achieved initial results. However, the vegetation coverage in the southern part of the study area is higher, but the soil erosion intensity is higher than that in other areas with the same coverage in the study area, which is due to the lack of understory vegetation and the loss of biodiversity, resulting in the decline in soil erosion resistance. Light erosion is mainly on the south and northwest sides. These areas are village ruins with weeds on the surface, and plant roots could control soil separation and loss, but their vegetation coverage is low and the soil is prone to rain splash erosion. Moderate erosion is mainly distributed in areas under construction in the north and west, and abandoned fish ponds in the west. Most of these areas have low or even no vegetation cover and are directly affected by heavy rains. Human activities are the primary cause of soil erosion in these areas.

The causes of soil erosion involve natural factors and human factors [58]. Natural factors are the objective potential conditions for the occurrence and progression of soil erosion. Improper human activities are the main factors that aggravate soil erosion. With the development of the city, industrial production and engineering construction are also accelerating, and further, the disturbance to land and vegetation is becoming increasingly intense. The ecology of Xuejiadao is more sensitive, so more attention should be paid to soil protection. Therefore, based on the current situation of soil erosion in this area, this study proposes some suggestions for soil and water conservation.

1. For areas with a large slope and bare surface, soil erosion can be effectively controlled by increasing barren slope treatment and afforestation.

2. The authorities should take action to transform a single pure forest into a multilayer mixed forest structure, increase understory vegetation, improve forest community ecological function, and improve the ability of water and soil conservation, such as establishing a coniferous mixed forest and tree shrub complex forest.

3. Saline-tolerant plants are planted to increase the organic matter content of the soil as much as possible, improve the physicochemical properties of the soil, and reduce the impact of soil salinization on soil erosion.

4. In important ecologically fragile and sensitive areas, industrial production and construction activities that may cause soil erosion shall be restricted or prohibited.

5. Industrial production and construction activities ensure that soil and water conservation measures are implemented. Supervisory and administrative departments could make full use of remote sensing, UAV, information networks, and so on to strengthen the monitoring of soil and water conservation.

## 5. Conclusions

Due to rapid population growth, rapid socioeconomic development, the continuous progress of industrialization and urbanization, and global changes, the soil has begun to degrade. With the improvement of human environmental awareness, some measures have been carried out, such as afforestation and planting grass, greening barren mountains and so on, to improve the ecological environment. However, the impact of planted forests on soil erosion is different from that of natural forests. Traditional soil erosion research methods are mostly based on satellite remote sensing data, which cannot accurately represent the erosion status of planted forests. In addition, traditional soil erosion models are unable to characterize the complex relationships between the influencing factors. Therefore, the purpose of this paper is to accurately understand the soil erosion of planted forests by classifying and identifying the soil erosion intensity in the southern part of Xuejiadao, so as to control the soil erosion in the ecological restoration area in a more targeted manner. Based on the multi-spectral image and elevation data obtained by UAV, as well as rainfall and soil, this paper constructed a CNN model to classify soil erosion intensity in the southern part of Xuejiadao in 2021. Furthermore, the identification results of CNN were compared and analyzed with those of the traditional method. In this study area, the accuracy of the CNN was improved by 25.57% compared to the traditional method of soil erosion study. The accuracy of the CNN in predicting the soil erosion intensity in the ecological restoration area was higher. In addition, the effects of slope, land use type, and vegetation cover on soil erosion were analyzed. lt was found that the soil erosion in the studied area was predominantly slight, and the erosion area also occurred in places with less vegetation cover and steep slopes. In view of this, this paper summarizes five suggestions to strengthen the prevention and control of soil erosion.

The results could provide a scientific basis for soil erosion control and ecological environment improvement in the study area. At the same time, it also provides a good template for soil erosion research in other similar areas. However, UAV images need to be collected multiple times, and the data obtained under different flight conditions could be different, which may lead to minor differences in the practical modeling approach. In addition, the study area is too small to reflect the impact of rainfall changes on soil erosion and soil erosion varies from region to region. CNN’s classification and recognition of soil erosion require further research in different places to achieve more precise predictions. In addition, this paper only makes a fine identification of soil erosion intensity but fails to quantitatively analyze soil erosion which is also a challenge for both data collection and modeling for future research.

## Figures and Tables

**Table 1:** UAV-related parameters and settings.

Name	Related Parameters
UAV model	DJI Phantom 4 Multispectral
Flight altitude	Relative altitude 100 m
Aerial photography accuracy	0.05 m
Flight speed	25.1 km/h
Photo interval	2 s
Hovering accuracy	Enable and normal operation: vertical: ±0.1 m; horizontal: ±0.1 m
Flight time	10:00~14:00
Shooting route	“Bow” type
Heading overlap rate	80%
Bypass overlap rate	70%
Flight duration	Approximately 20 min for a single flight Total flight time 20 h
Total flight distance	460 km
Photo shoot	148,680 sheets
Image Resolution	Each image has a resolution of 1600 × 1300 pixels, with an overall output of 80,038 × 91,959 pixels

**Table 2:** P values of different land use types in the study area.

Land Use Classification	Woodland	Shrubland	Grassland	Construction Land	Waters	Bare Land
P value	0.2	0.4	0.7	0	0	0.9

**Table 3:** Calculation results of model evaluation index.

Models (Accuracy)	Indicators	Slight	Light	Moderate
RUSLE (70.33%)	Recall	70.33%	0.00%	0.00%
	Precision	100.00%	0.00%	0.00%
	F1-Score	41.28%	0.00%	0.00%
SVM (90.63%)	Recall	91.75%	87.86%	92.11%
	Precision	91.85%	85.49%	94.88%
	F1-Score	91.80%	86.66%	93.47%
FNN (92.00%)	Recall	90.02%	88.96%	91.09%
	Precision	86.14%	89.07%	100.00%
	F1-Score	88.04%	89.01%	95.34%
CNN (95.90%)	Recall	96.29%	94.64%	96.64%
	Precision	95.58%	95.12%	97.14%
	F1-Score	95.89%	94.85%	96.90%

**Table 4:** Soil erosion intensity of different land use types.

Land-Use	Area (hm^2^)	Area Ratio (%)		
		Slight	Light	Moderate
Waters	24.71	97.26	2.74	0.00
Grassland	317.53	48.01	37.30	14.69
Shrubland	104.02	86.64	10.54	2.82
Woodland	111.07	87.56	9.60	2.84
Construction land	30.49	95.07	4.93	0.00
Bare land	163.20	23.75	17.35	58.90

**Table 5:** Soil erosion intensity of different vegetation coverages.

Vegetation Coverage	Area (hm^2^)	Area Ratio (%)		
		Slight	Light	Moderate
<10%	109.95	39.28	15.23	45.49
10~30%	58.88	36.22	20.03	43.75
30~50%	63.46	37.04	25.92	37.04
50~70%	89.15	43.81	34.54	21.65
≥70%	429.58	66.00	22.90	11.10

**Table 6:** Soil erosion intensity at different slopes.

Slope	Area (hm^2^)	Area Ratio (%)		
		Slight	Light	Moderate
≤2${}^{\circ }$	255.80	0.47	0.25	0.27
2~6${}^{\circ }$	294.70	0.61	0.21	0.18
6~15${}^{\circ }$	170.03	0.56	0.25	0.19
15~25${}^{\circ }$	23.13	0.55	0.15	0.30
>25${}^{\circ }$	7.36	0.29	0.20	0.51

## Data Availability

The raw data supporting the conclusions of this article will be made available on request from the corresponding author, without undue reservation.
